# Elimination of Epidemic Methicillin-Resistant *Staphylococcus aureus* from a University Hospital and District Institutions, Finland

**DOI:** 10.3201/eid0902.020233

**Published:** 2003-02

**Authors:** Pirkko Kotilainen, Marianne Routamaa, Reijo Peltonen, Jarmo Oksi, Esa Rintala, Olli Meurman, Olli-Pekka Lehtonen, Erkki Eerola, Saara Salmenlinna, Jaana Vuopio-Varkila, Tuire Rossi

**Affiliations:** * Turku University Central Hospital, Turku, Finland; † National Public Health Institute, Turku, Finland; ‡ Satakunta Central Hospital, Pori, Finland; § Turku University, Turku, Finland; ¶ National Public Health Institute, Helsinki, Finland

**Keywords:** MRSA, multiresistant bacteria, hospital outbreaks, MRSA control policy, research

## Abstract

From August 1991 to October 1992, two successive outbreaks of methicillin-resistant *Staphylococcus aureus* (MRSA) occurred at a hospital in Finland. During and after these outbreaks, MRSA was diagnosed in 202 persons in our medical district; >100 cases involved epidemic MRSA. When control policies failed to stop the epidemic, more aggressive measures were taken, including continuous staff education, contact isolation for MRSA-positive patients, systematic screening for persons exposed to MRSA, cohort nursing of MRSA-positive and MRSA-exposed patients in epidemic situations, and perception of the 30 medical institutions in that district as one epidemiologic entity brought under surveillance and control of the infection control team of Turku University Hospital. Two major epidemic strains, as well as eight additional strains, were eliminated; we were also able to prevent nosocomial spread of other MRSA strains. Our data show that controlling MRSA is possible if strict measures are taken before the organism becomes endemic. Similar control policies may be successful for dealing with new strains of multiresistant bacteria, such as vancomycin-resistant strains of *S. aureus*.

Methicillin-resistant *Staphylococcus aureus* (MRSA) has emerged worldwide as an important nosocomial pathogen. In some U.S. hospitals, MRSA already accounts for 30% to 50% of all nosocomial *S. aureus* isolates. The situation is comparable in many European centers: according to a recent survey (*1*), the proportion of MRSA compared to all nosocomial *S. aureus* isolates studied was >50% in Portugal and Italy and >30% in Turkey and Greece. The methicillin-resistance rate was low (2.0%) in the Netherlands, calling attention to the distinguished Dutch MRSA strategy (*2*). Switzerland, which had the lowest MRSA prevalence (1.8%) in the European survey (*1*), is noted for innovative interventions to improve hand hygiene in hospitals and, thereby, to reduce MRSA transmission (*3*).

In the Scandinavian countries, methicillin-resistant strains still account for <1% of all nosocomial *S. aureus* isolates (*4*). MRSA has remained uncommon also in Finland (*5*,*6*), and until the 1990s, mostly sporadic cases of MRSA were identified in hospitalized patients. In recent years, however, several nosocomial outbreaks caused by different epidemic strains have occurred (*6*). Two successive MRSA outbreaks at the Turku University Hospital, Finland, and in nearby institutions were the first and, so far, the second largest. We describe the Turku outbreaks and the subsequent yearly numbers of new MRSA cases identified in our district. We also discuss the control measures taken, which have been followed since then, to confine the spread of epidemic MRSA at the university hospital and in the whole Southwest Finland Medical District.

## Methods

### Background

The Turku University Hospital is a teaching facility that serves as a tertiary referral center for southwestern Finland. Approximately 500,000 inhabitants live in the Southwest Finland Medical District; the density of the population varies from 20–100 inhabitants per square kilometer. The institutions include 1 university hospital, 1 central hospital, 7 regional hospitals, and 22 health-care centers.

From August 1991 to October 1992, two successive outbreaks caused by different MRSA strains occurred in the departments of surgery and medicine at University Hospital. During and after these outbreaks, these two MRSA strains were isolated from patients and staff members in five additional institutions in our district.

### Screening Policy

Our policy of screening contact patients of the MRSA-positive patients and the hospital staff for MRSA varied during the different phases of the outbreaks. Unless otherwise indicated, the term contact patient refers to a patient hospitalized on the same ward at the same time with an MRSA-positive patient.

### Surgical Unit Outbreak

During the outbreak in the surgical unit, in most cases MRSA was isolated from a clinical specimen. Initially, a policy decision was made not to screen either the contact patients of MRSA-positive patients or the staff on outbreak wards for MRSA. When the number of MRSA cases increased, we performed one cross-sectional study to screen all patients cared for in the department of surgery during that particular day for nasal and wound colonization by MRSA.

### Medical Unit Outbreak

After the first two cases were diagnosed in the medical unit, we began screening other patients treated in the medical intensive-care unit (ICU). During weeks 6–8, all contact patients connected with MRSA-positive patients (treated in the ICU after admission of the index case) were screened once by using nasal swabs if they were still hospitalized. If a new case was identified on a ward, the roommates of the patient were screened. If transmission of MRSA was observed on a ward, all patients were screened. Initially, screening involved only nasal swabbing, but from the first week of June 1992 on, cultures were taken also from the perineum, groin, and axillae, as well as from all open wounds, skin lesions, and, later, the throat. After 10 identified cases of MRSA, we began to label case records of the colonized patients and contact patients with tags showing MRSA information. The contact patients were screened on the next visit; previous roommates of MRSA-positive patients were isolated while waiting for culture results. After providing two sets of negative MRSA cultures, contact patients were no longer screened on subsequent admissions. However, patients once found to be MRSA positive were screened on subsequent admissions and placed in single rooms to be cared for in contact isolation. All patients previously treated at hospitals abroad or with a known MRSA problem were screened at the time of admission and nursed in contact isolation until results from colonization cultures were negative. We screened the staffs of the medical ICU and the hematology and infectious diseases units by nasal swab at varying intervals during the medical outbreak. Screening cultures were done, as described (*7*,*8*).

### Identification of MRSA

The isolates grown on culture plates were identified as MRSA following the National Committee for Clinical Laboratory Standards guidelines (*9*). Genetic resistance to methicillin was verified by the presence of the *mecA* gene (*10*). All MRSA isolates were submitted to the Staphylococcal Reference Laboratory at the National Public Health Institute, where they were typed with the international phage set and ribotyping and pulsed-field gel electrophoresis (PFGE), as described (*6*). Two isolates were defined as different strains if they had different phage types and/or PFGE and ribotypes. We considered phage types different if two or more strong phage reaction differences occurred. Ribotypes were considered different if any band difference occurred. Before 1995, PFGE types were considered different if any band difference occurred. After 1995, PFGE types were considered different if four or more band differences occurred.

### Elimination Treatment

Elimination treatment with topical or combined topical and systemic antimicrobial therapy was given to selected patients (e.g., long-term care patients of health-center wards and nursing homes) and those with severe underlying diseases who were frequently admitted to any hospital in the district (*7*,*8*,*11*). Long-term carriers among the staff were also given elimination treatment. Detailed data on drug regimens will be reported separately.

## Results

### MRSA Strains

From 1991 through 2000, a total of 202 persons in the Southwest Finland Medical District were infected or colonized by MRSA (Table). On the basis of phage typing and molecular typing, we identified 15 different MRSA strains isolated from two or more persons. These strains included 10 isolated from hospitalized patients (outbreak strains) and 5 causing intrafamilial clusters in the community (familial strains). The strain causing the surgical outbreak (referred to as the surgical strain) belonged to phage type 75,77,84,85III and had a characteristic ribotype and PFGE pattern. The strain causing the medical outbreak (referred to as the medical strain) was nontypable with phages (NT), but the strain relatedness between different isolates could be ascertained by ribotyping and PFGE. A third MRSA strain typed 54,84,85III/96V/95 caused the Mynamaki Health Center outbreak described previously (*7*). A detailed typing analysis, including a picture of the PFGE profiles of these major epidemic strains, has been published (*6*) and describes the corresponding strain identification code as E6 for the surgical strain, E7 for the medical strain, and O9 for the Mynamaki strain. The cases involved in these three outbreaks, as well as the clusters caused by 12 additional MRSA strains, are summarized in the Table. The remaining 63 strains were isolated from one person each.

**Table:** Yearly number of new cases caused by different methicillin-resistant *Staphylococcus aureus* (MRSA) strains, Southwest Finland Medical District, 1991–2000

Year	Strains								
	Surgical outbreak^a^	Medical outbreak^b^	Mynamaki outbreak^c^	MRSA outbreak IV^d^	MRSA outbreak V^e^	Other outbreaks	Familial MRSA^f^	Solitary MRSA	Total
1991	11							2	13
1992	19	56				2^g^		1	78
1993	1	1	13	4		2^h^	2	4	27
1994							3	2	5
1995	4							3	7
1996	2							7	9
1997							2	6	8
1998						2^g^		17	19
1999						2^g^	2	10	14
2000					5	2^g^	4	11	22
Total	37	57	13	4	5	10	13	63	202

^a^Strain was recovered from 24 patients at the university hospital,10 patients at a regional hospital, and 3 staff members in these hospitals.

^b^Strain was recovered from 30 patients and 18 staff members at the university hospital and from 9 patients in other district institutions.

^c^Strain was recovered from 12 patients and 1 staff member in a health-center ward and associated nursing home (*7*).

^d^Strain caused a cluster of four cases in an intensive-care unit of a central hospital.

^e^Strain caused a cluster of four infected patients and one infected staff member in a health-center ward at the beginning of 2000, but was subsequently eliminated.

^f^Intrafamilial clusters of two to four MRSA cases in the community.

^g^MRSA strain was transmitted from one patient to another at the university hospital.

^h^MRSA strain was transmitted from one patient to another at a regional hospital.

Three (30%) of 10 outbreak strains and 22 (35%) of 63 unique strains were designated as of foreign origin. None of the five familial strains were of foreign origin.

### MRSA Outbreaks at the University Hospital

#### Surgical Unit Outbreak

**Figure 1:**
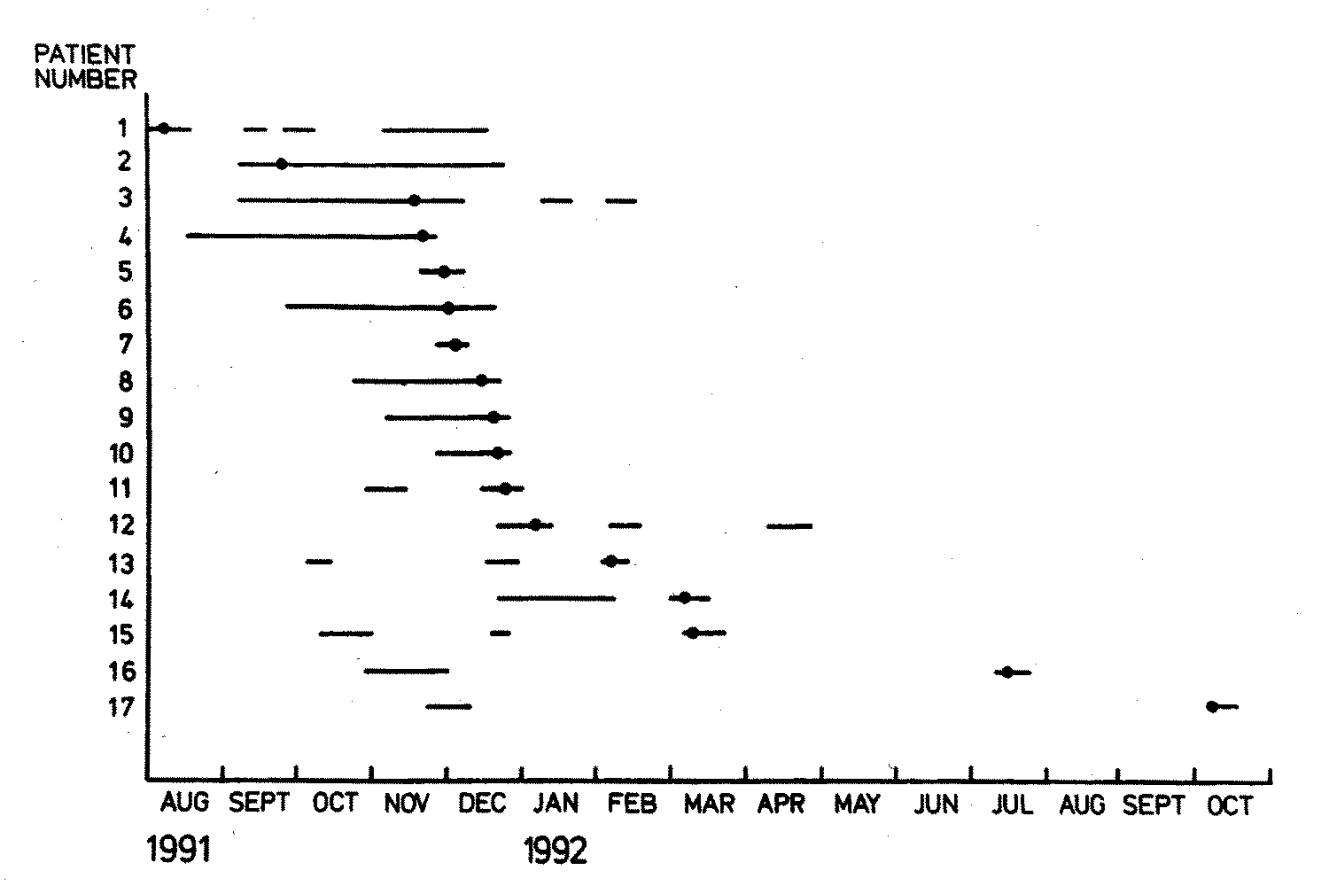
Spread of surgical outbreak strain. Methicillin-resistant *Staphylococcus aureus* (MRSA) isolated August 1991–October 1992 in 17 patients cared for on two surgical wards and the surgical intensive-care unit. Hospitalization periods of these patients are shown as horizontal lines. Symbol • indicates the time point when the first culture positive for MRSA was taken.

The hospitalization periods of the patients during the surgical outbreak and the times when MRSA was first isolated in each case are shown in Figure 1. In August 1991, the surgical strain was isolated from a bone sample of patient 1 who was cared for on an orthopedic ward for posttraumatic osteomyelitis. The patient was referred to the infectious diseases unit to be cared for in contact isolation, but she was readmitted to the orthopedic ward three times during the following 4 months for treatment of osteomyelitis. Each time, the isolation precautions followed by hospital personnel did not comply with the standard adopted later.

MRSA was next isolated from head wound of a colonized male patient on the same ward. He was placed in a single room to be cared for in contact isolation, but when the wound healed, the patient was transferred to a three-bed room. Subsequently, three of his roommates (patients 3, 4, and 5) acquired MRSA. By the 3rd week of December 1991, the combined number of patients colonized by epidemic MRSA had increased to eight cases on two wards and in the surgical ICU. A shortage of single rooms and the threat of an expanding outbreak led to implementation of the following control measures: 1) intensive education of the staff on hospital hygiene, 2) nursing of all MRSA-positive patients in single rooms in contact isolation, preferably in the infectious diseases unit, 3) strict adherence to contact isolation precautions and minimal duration of hospitalization whenever an MRSA-positive patient was treated at the department of surgery (e.g., operative treatment required), and 4) cross-sectional screening of all patients nursed on surgical wards and in the surgical ICU on December 19, 1991, for nasal and wound colonization. The screening uncovered three new cases of MRSA on epidemic wards. By year end, all patients identified as MRSA positive had been either discharged or transferred to the infectious diseases unit. Thereafter, no new transmission of MRSA was observed on surgical wards, although by the end of August 1993, the surgical strain was isolated from clinical specimens of eight additional patients who had been cared for on the epidemic wards during 1991–1992. These patients had evidently acquired the surgical strain while hospitalized during the outbreak, but the MRSA colonization was not recognized then because screening was not done routinely.

In November 1995, the surgical strain was unexpectedly isolated from an endotracheal aspirate of a patient in the surgical ICU. This patient had also been cared for on the orthopedic ward during the 1991 outbreak. Subsequent screening of contact patients in the ICU showed MRSA colonization in three other patients who had ventilatory support at the same time. No new transmission of MRSA was observed after these patients were transferred to the infectious diseases unit. The total number of University Hospital patients infected or colonized by the surgical strain was 24.

#### Medical Unit Outbreak

The index patient was treated for cerebral hemorrhage in an ICU in Rome, Italy. After his referral to the department of neurology of University Hospital in December 1991, the medical strain was isolated from his endotracheal aspirate. For the next 3 months, the patient was cared for in contact isolation in a single room on a neurologic ward; we found no evidence of MRSA transmission to other patients on that ward.

#### Medical ICU

In March 1992, the index patient became ill with septic shock caused by MRSA and was admitted to the medical ICU for respiratory support. For the first 24 hours, he was not isolated because of a misunderstanding but treated in the same room with three other patients who had ventilatory support. His contact patients were neither screened nor isolated.

**Figure 2:**
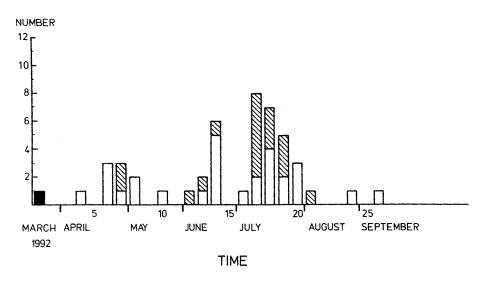
Number of new cases weekly of methicillin-resistant *Staphylococcus aureus* among patients and staff during the medical outbreak, third week of March to the second week of September 1992. Time is shown in weeks from the admission of the index case (black column) to the medical intensive-care unit. White columns indicate patient cases; striped columns indicate staff cases.

Two weeks later (Figure 2), the medical strain was cultured from an endotracheal aspirate of a patient who had died in the ICU a few days earlier. The devised screening program was delayed, and subsequent screening on weeks 6–8 found six new patient carriers and two staff carriers of MRSA. The medical ICU was closed to new admissions, and an auxiliary ICU was established for those patients who had not been exposed to MRSA.

In the auxiliary ICU, a new staff carrier of MRSA was identified on week 12 and a new patient carrier and a staff carrier on week 13. The MRSA-positive patient was immediately referred to the infectious diseases unit. The six other patients who had shared the ICU room with him were simultaneously transferred to that same unit. Screening cultures later revealed MRSA colonization in five of them, indicating that early cohorting of these contact patients may have been critical in preventing further spread.

#### Hematology Unit

In May 1992, we identified MRSA colonization in four patients cared for on the hematology ward. Two of them became colonized while being treated in the medical ICU in April and transmitted MRSA to their two roommates on the ward before carriage became manifest. Using nasal swabs, we screened a number of patients treated at that time on the same ward. Many other contact patients already discharged were not screened when they were readmitted, rendering further spread of MRSA possible. At the beginning of July 1992, MRSA was isolated from an endotracheal aspirate of a bone marrow transplant patient cared for on the hematology ward (Figure 2). Subsequent screening showed colonization in 11 additional hematologic patients and 12 staff members.

We prevented nosocomial transmission by immediately closing the hematology ward. For the next 3 months, hematologic patients were cared for in three separate cohorts: 1) those not exposed to MRSA were admitted to the hematology unit when it was reopened, 2) those potentially exposed to MRSA during the previous 4 months were cared for in a separate cohort in the infectious diseases unit until three sets of colonization cultures had proved negative, and 3) those colonized by MRSA were cared for in the infectious diseases unit. The total number of University Hospital patients colonized by the medical strain was 30, and the last case was identified in February 1993. This patient had evidently become colonized in April 1992 while being treated in the ICU at the same time as the index case. His MRSA colonization had remained unknown, since contact patients were not screened at that time.

#### Staff Carriage

A total of 20 staff members were colonized with MRSA during these two outbreaks. All five long-term carriers received elimination treatment with a successful outcome. The staff members who were colonized were sent home but could return to work after they had provided three successive negative MRSA cultures.

### MRSA in Other District Institutions

In August 1992, the first case of the surgical strain was identified at Turku City Hospital. Subsequent screening found colonization in seven additional patients on three different wards. After two more cases were identified in 1996, the total number of city hospital patients colonized by the surgical strain was 10. During August and September 1992, we found nine patients in two local hospitals and two health-center wards colonized by the medical strain. In each institution, MRSA was first isolated from a clinical sample, and screening of contact patients on the ward found a few additional cases. The infection control team of University Hospital visited each facility to delineate appropriate control measures for MRSA. Colonized patients were referred to the infectious diseases unit for elimination treatment. Other patients were screened, and those found to be colonized were cared for in contact isolation until they could be admitted to the infectious diseases unit for decolonization. By following this strict control policy, we were able to eliminate MRSA from these five institutions.

In 1993, the Mynamaki Health Center outbreak was controlled as previously described after 13 cases (*7*), and a central hospital ICU outbreak was controlled after four cases (outbreak strain IV). In 2000, MRSA outbreak strain V was eliminated from a long-term care facility after five cases occurred. Of the five additional epidemic MRSA strains, one was eliminated after two cases in a regional hospital, and the other four strains were eliminated after causing two cases each at University Hospital (Table).

### Long-Term Follow-Up of Patients

Of the 37 patients who acquired the surgical strain, 6 died within 1 month after MRSA was identified, 20 died during the following years, and 3 were not part of follow-up. Eight patients remain residents in our district, two of them still carrying the surgical strain. The majority of the 39 patients who acquired the medical strain had severe underlying diseases. Of all 39 patients, 21 died within 3 months of colonization, 12 died during the following years, and 1 was not part of follow-up. Five patients who still live in Turku, three of whom were treated to eliminate carriage, no longer carry MRSA. Thus, the medical strain has been eliminated from our district.

## Discussion

During past few years, news on MRSA has usually been discouraging. Clinicians and infection control practitioners appear to have lost confidence in their capability to control the nosocomial spread of this pathogen. The number of papers focusing on the overwhelming spread of MRSA is increasing (*1*,*12*–*17*), whereas those addressing successful efforts of control or stating that nosocomial spread of MRSA can and should be controlled are few (*18*–*22*). A number of researchers debating the control of MRSA have questioned whether controlling this microorganism is reasonable, feasible, or justified (*23*–*25*) and whether the tracing of colonized people is justified (*26*). We describe the elimination of MRSA from a university hospital and a medical-district–wide control policy for MRSA after the outbreak. Our results show that controlling or even eliminating MRSA is possible, if strict measures are systematically taken before the organism becomes endemic. Our experience should encourage other countries with a low incidence of MRSA to continue efforts to prevent the spread of this microorganism in hospitals and long-term care facilities.

According to 46 published reports on outbreaks, 10% of the hospitals with >40 cases have achieved definite or probable elimination of MRSA (*27*). Although >100 patients and staff members in our district initially became colonized by epidemic MRSA, this microbe is being controlled almost 10 years after these first outbreaks. We eliminated the medical strain from the whole district, and only a few outpatients presently carry MRSA in the community. Moreover, we were able to prevent nosocomial spread of the almost 100 additional MRSA strains encountered in our area. Even the 22 MRSA strains introduced by patients transferred from hospitals abroad have remained solitary cases, despite their epidemic potential. In fact, after the small university hospital ICU outbreak in 1995, nosocomial transmission of MRSA has been detected in our district hospitals only three times; on each occasion, MRSA colonization of the index case was not known or suspected on admission.

Containment of the Turku outbreaks in 1991–1992 was greatly impeded by the fact that we had no national guidelines on how to control MRSA in Finland at that time and very little previous experience with these microorganisms. Detailed guidelines published by authorities from abroad advised an active control policy (*28*), but stringent measures were perceived by our colleagues as too disruptive for the patient care in our institution. One major argument against adopting an aggressive line of control was the lack of severe MRSA infections because many of our patients were colonized without clinical infection. During the early phase of the medical outbreak, the infection control team adjusted to a lenient control policy because of our previous experience with the surgical strain, which was easily contained. The behavior of the medical strain, however, was quite different from the surgical strain, and the inadequacy of the control measures at the beginning of the medical outbreak is now evident. We may have been able to restrict this outbreak to only a few cases if all ICU patients had been screened for MRSA and MRSA-positive patients had been isolated as soon as we discerned that appropriate control measures had not been taken when caring for the index patient or if the medical ICU had been closed to new admissions after the second or third MRSA case. Similarly, screening of only some of the MRSA contact patients in the hematology unit in May 1992 was clearly insufficient. Had our efforts initially been more aggressive and the outbreaks quickly controlled, we may have saved many persons from becoming colonized with MRSA and considerably reduced the costs of infection control measures required.

The most important lesson from these first epidemics was that an ambivalent and permissive control policy for MRSA easily fails. We have subsequently made every effort to avoid making the same mistake. Whenever MRSA has been introduced into our hospitals, rapid steps have been taken to adopt appropriate control measures. The mainstays of our present policy involve continuous staff education, caring for MRSA-positive patients in single rooms in contact isolation, systematic screening of patients exposed to MRSA, including all patients transferred from hospitals abroad or with a known MRSA problem, and cohort nursing of MRSA-positive and exposed patients, at least in epidemic situations.

National guidelines have proved most beneficial in preventing the spread of MRSA in a few low-incidence countries (*2*,*6*). Medical-district–wide guidelines may be equally important when an individual hospital is struggling with MRSA and needs practical or moral support. The Turku MRSA policy involves perceiving our medical district with its approximately 30 institutions as one epidemiologic entity; the infection control team of University Hospital is responsible for the control of MRSA (and also of other multiresistant bacteria) in the whole entity. This overall responsibility ensures that the same control policy for MRSA is followed in all district institutions. If MRSA is encountered in any local hospital or health-center ward, consultation is given; if nosocomial transmission of the microbe is observed, the infection control team visits the institute. We continuously strive to prevent the development of MRSA reservoirs in our extended-care facilities. In so doing, treatment to eliminate MRSA carriage in long-term patients has been favored, while in contrast, efforts to eliminate MRSA in outpatients have not had the same focus.

Many of our experiences were taken into account when the National Guidelines for the Control of MRSA in Finland were prepared in 1995 (*6*). To a great extent, the MRSA control policy finally adopted is in line with that currently followed in the Netherlands (*2*) and initially recommended by the British authorities in 1990 (*28*). Because of the increasing prevalence of MRSA, those guidelines were replaced in the U.K. by more lenient instructions in 1998 because the situation in many parts of the country was such that a more flexible approach was considered appropriate (*29*,*30*). With the dramatic increase of MRSA, other countries (including the United States) where these microbes are already endemic in hospitals have adopted more flexible control policies (*31*,*32*). However, now that vancomycin-resistant *S. aureus* (VRSA) exists (*33*), controlling MRSA is even more imperative. A lenient or ambivalent policy is especially inappropriate in those countries where MRSA remains uncommon, since they may still have a fair chance of eliminating this pathogen. In southwestern Finland, the factors possibly contributing to our success include active education and excellent compliance of health-care personnel, a uniform health-care system, and low population density.

Anticipating the emergence of new and even more serious strains of multiresistant staphylococci poses a demanding challenge to clinicians and infection-control practitioners worldwide to seek novel methods, which could effectively prevent the spread of these microorganisms. Despite an inability to control MRSA in many countries, we believe that confining these newly emerging multiresistant strains may be possible, provided that vigorous efforts are taken early while the microbe still remains rare. If rapidly begun, aggressive measures may not be needed for long and thereby be cost-effective. To meet future challenges successfully, a stringent and consistent international control policy should be issued and universally obeyed.

We have shown that controlling or even eliminating MRSA is possible, if strict measures are taken before the organism becomes endemic. A similar policy may be successful when combating new and even more serious strains of multiresistant bacteria (e.g., VRSA). The recent emergence of VRSA emphasizes the need for unremitting and vigorous control of MRSA. National guidelines for MRSA control policy have proven beneficial in a few low-incidence countries. Our results suggest that firm international guidelines will aid countries in preventing the global spread of any newly emerging multiresistant bacterial pathogen. An ultimate prerequisite for success is the commitment of the health-care personnel worldwide to struggle for that important goal.

## References

[1] Diekema DJ, Pfaller MA, Schmitz FJ, Smayevsky J, Bell J, Jones RN, Survey of infections due to *Staphylococcus* species: frequency of occurrence and antimicrobial susceptibility of isolates collected in the United States, Canada, Latin America, Europe, and the Western Pacific Region for the SENTRY antimicrobial surveillance program, 1997–1999. Clin Infect Dis. 2001;32(Suppl 2):114–32. 10.1086/320184 11320452

[2] Verhoef J, Beaujean D, Blok H, Baars A, Meyler A, van der Werken C, A Dutch approach to methicillin-resistant *Staphylococcus aureus.* Eur J Clin Microbiol Infect Dis. 1999;18:461–6. 10.1007/s100960050324 10482021

[3] Pittet D, Hugonnet S, Harbarth S, Mourouga P, Sauvan V, Touveneau S, Effectiveness of a hospital-wide programme to improve compliance with hand hygiene. Lancet. 2000;356:1307–12. 10.1016/S0140-6736(00)02814-2 11073019

[4] Voss A, Milatovic D, Wallrauch-Schwarz C, Rosdahl VT, Braveny I. Methicillin-resistant *Staphylococcus aureus* in Europe. Eur J Clin Microbiol Infect Dis. 1994;13:50–5. 10.1007/BF02026127 8168564

[5] Hyvärinen J, Huovinen P, Järvinen H, Kotilainen P. Multiresistance in *Staphylococcus* spp. blood isolates in Finland with special reference to the distribution of the *mec*A gene among the *Staphylococcus epidermidis* isolates. APMIS. 1995;103:885–91. 10.1111/j.1699-0463.1995.tb01448.x 8562029

[6] Salmenlinna S, Lyytikäinen O, Kotilainen P, Scotford R, Siren E, Vuopio-Varkila J. Molecular epidemiology of methicillin-resistant *Staphylococcus aureus* in Finland. Eur J Clin Microbiol Infect Dis. 2000;19:101–7. 10.1007/s100960050438 10746495

[7] Kotilainen P, Routamaa M, Peltonen R, Evesti P, Eerola E, Salmenlinna S, Eradication of methicillin-resistant *Staphylococcus aureus* from a health center ward and associated nursing home. Arch Intern Med. 2001;161:859–63. 10.1001/archinte.161.6.859 11268229

[8] Rossi T, Peltonen R, Laine J, Eerola E, Vuopio-Varkila J, Kotilainen P. Eradication of the long-term carriage of methicillin-resistant *Staphylococcus aureus* in patients wearing dentures: a follow-up of 10 patients. J Hosp Infect. 1996;34:311–20. 10.1016/S0195-6701(96)90111-5 8971620

[9] National Committee for Clinical Laboratory Standards. Performance standards for antimicrobial disc susceptibility tests. Villanova (PA): The Committee; 1990. Document M2–A4.

[10] Predari SC, Ligozzi M, Fontana R. Genotypic identification of methicillin-resistant coagulase-negative staphylococci by polymerase chain reaction. Antimicrob Agents Chemother. 1991;35:2568–73. 1810190 10.1128/aac.35.12.2568 PMC245432

[11] Rossi T, Laine J, Eerola E, Kotilainen P, Peltonen R. Denture carriage of methicillin-resistant *Staphylococcus aureus* [letter]. Lancet. 1995;345:1577. 10.1016/S0140-6736(95)91129-4 7791467

[12] Aires de Sousa M, Santos Sanches I, Ferro ML, Vaz MJ, Saraiva Z, Tendeiro T, Intercontinental spread of a multidrug-resistant methicillin-resistant *Staphylococcus aureus* clone. J Clin Microbiol. 1998;36:2590–6. 9705398 10.1128/jcm.36.9.2590-2596.1998 PMC105168

[13] Couto I, Melo-Cristino J, Fernandes ML, Garcia T, Serrano N, Salgado MJ, Unusually large number of methicillin-resistant *Staphylococcus aureus* clones in a Portuguese hospital. J Clin Microbiol. 1995;33:2032–5. 7559943 10.1128/jcm.33.8.2032-2035.1995 PMC228330

[14] Farrington M, Redpath C, Trundle C, Coomber S, Brown NM. Winning the battle but losing the war: methicillin-resistant *Staphylococcus aureus* (MRSA) infection at a teaching hospital. QJM. 1998;91:539–48. 10.1093/qjmed/91.8.539 9893757

[15] The Hopital Propre II Study Group. Methicillin-resistant *Staphylococcus aureus* in French hospitals: a 2-month survey in 43 hospitals, 1995. Infect Control Hosp Epidemiol. 1999;20:478–86. 10.1086/501656 10432160

[16] de Lencastre H, Severina E, Milch H, Konkoly Thege M, Tomasz A. Wide geographic distribution of a unique methicillin-resistant *Staphylococcus aureus* clone in Hungarian hospitals. Clin Microbiol Infect. 1997;3:289–96. 10.1111/j.1469-0691.1997.tb00616.x 11864123

[17] Aucken HM, Ganner M, Murchan S, Cookson BD, Johnson AP. A new UK strain of epidemic methicillin-resistant *Staphylococcus aureus* (EMRSA-17) resistant to multiple antibiotics. J Antimicrob Chemother. 2002;50:171–5. 10.1093/jac/dkf117 12161396

[18] Dancer SJ, Crawford A. Keeping MRSA out of a district general hospital. J Hosp Infect. 1999;43(Suppl):19–27. 10.1016/S0195-6701(99)90062-2 10658755

[19] Herwaldt LA. Control of methicillin-resistant *Staphylococcus aureus* in the hospital setting. Am J Med. 1999;106:11–8. 10.1016/S0002-9343(98)00350-7 10348059

[20] Vandenbroucke-Grauls CM, Frenay HME, van Klingeren B, Savelkoul TF, Verhoef J. Control of epidemic methicillin-resistant *Staphylococcus aureus* in a Dutch university hospital. Eur J Clin Microbiol Infect Dis. 1991;10:6–11. 10.1007/BF01967090 2009885

[21] Jernigan JA, Titus MG, Groschel DH, Getchell-White S, Farr BM. Effectiveness of contact isolation during a hospital outbreak of methicillin-resistant *Staphylococcus aureus.* Am J Epidemiol. 1996;143:496–504. 8610665 10.1093/oxfordjournals.aje.a008770

[22] Karchmer TB, Durbin LJ, Simonton BM, Farr BM. Cost-effectiveness of active surveillance cultures and contact/droplet precautions for control of methicillin-resistant *Staphylococcus aureus.* J Hosp Infect. 2002;51:126–32. 10.1053/jhin.2002.1200 12090800

[23] Barrett SP, Mummery RV, Chattopadhyay B. Trying to control MRSA causes more problems than it solves. J Hosp Infect. 1998;39:85–93. 10.1016/S0195-6701(98)90322-X 9651853

[24] Bowler ICJ. Is control of methicillin-resistant *Staphylococcus aureus* justified? QJM. 1997;90:243–6. 10.1093/qjmed/90.4.243 9307757

[25] Pittet D, Waldvogel FA. To control or not to control colonization with MRSA...that's the question. QJM. 1997;90:239–41. 10.1093/qjmed/90.4.239 9307756

[26] Teare EL, Barrett SP. Controversies: is it time to stop searching for MRSA? Stop the ritual of tracing colonised people. BMJ. 1997;314:665. 9066485 10.1136/bmj.314.7081.665 PMC2126093

[27] Boyce JM. Should we vigorously try to contain and control methicillin-resistant *Staphylococcus aureus?* Infect Control Hosp Epidemiol. 1991;12:46–54. 10.1086/646237 1999643

[28] Working Party report. Revised guidelines for the control of epidemic methicillin-resistant *Staphylococcus aureus.* J Hosp Infect. 1990;16:351–77. 10.1016/0195-6701(90)90008-C 1980508

[29] Ayliffe GA, Buckles A, Casewell MW, Cookson BD, Cox RA, Duckworth GJ, Revised guidelines for control of MRSA: applying appropriately-based recommendations. J Hosp Infect. 1999;43:315–6. 10.1016/S0195-6701(99)90429-2 10658809

[30] Working Party report. Revised guidelines for the control of methicillin-resistant *Staphylococcus aureus* infection in hospitals. J Hosp Infect. 1998;39:253–90. 10.1016/S0195-6701(98)90293-6 9749399

[31] Mulligan ME, Murray-Leisure KA, Ribner BS, Standiford HC, John JF, Korvick JA, Methicillin-resistant *Staphylococcus aureus*: a consensus review of the microbiology, pathogenesis, and epidemiology with implications for prevention and management. Am J Med. 1993;94:313–28. 10.1016/0002-9343(93)90063-U 8452155

[32] Wenzel RP, Reagan DR, Bertino JS, Baron EJ, Arias K. Methicillin-resistant *Staphylococcus aureus* outbreak: a consensus panel's definition and management guidelines. Am J Infect Control. 1998;26:102–10. 10.1016/S0196-6553(98)80029-1 9584803

[33] Centers for Disease Control and Prevention. *Staphylococcus aureus* resistant to vancomycin—United States, 2002. MMWR Morb Mortal Wkly Rep. 2002;51:565–7. 12139181

